# Laparoscopic hemostasis and drainage for postpartum retroperitoneal hematoma complicated with an infection: A case report and surgical video

**DOI:** 10.1016/j.amsu.2022.103686

**Published:** 2022-04-28

**Authors:** Takuya Yokoe, Masato Kita, Kentaro Suzuki, Yusuke Butsuhara, Aya Yoshida, Mamoru Morikawa, Hidetaka Okada

**Affiliations:** Department of Obstetrics and Gynecology, Kansai Medical University, 2-5-1 Sin-machi, Hirakata, Osaka, 573-1191, Japan

**Keywords:** Laparoscopic drainage, Laparoscopic hemostasis, Postpartum hematomas, Surgical film, Case report, CRP, C-reactive protein, CT, computed tomography, IVR, interventional radiology, MRI, magnetic resonance imaging, SCARE, surgical case report, WBC, white blood cell 1

## Abstract

**Introduction:**

and importance: Postpartum retroperitoneal hematomas are a potential complication of childbirth. The management of secondary infections of such hematomas has not been fully elucidated. We present a typical case of such management via laparoscopic surgery, and include a surgical video.

**Case presentation:**

A woman in her 20s experienced fever and right lower quadrant pain and distension on postpartum day 2. Pelvic examination revealed a hump on the vaginal wall on the right side of the uterine cervix, and ultrasonography revealed a hematoma. Contrast-enhanced computed tomography revealed no active extravasation into the hematoma. Conservative antibiotic treatment was started; however, on postpartum day 6, her pain increased and her C-reactive protein concentration and white blood cell count were high. Magnetic resonance imaging revealed a paravaginal/upper vaginal wall hematoma (80 × 70 × 63 mm) located to the right of the uterus and bladder. Hence, laparoscopic drainage was performed on postpartum day 7. The retroperitoneal hematoma was incised and drained. The source of bleeding was the right vaginal vein, and bleeding was halted via electrocoagulation. The patient's symptoms improved immediately, and the postoperative course was uneventful.

**Clinical discussion:**

The laparoscopic approach enabled immediate hemostasis and identification of the source of bleeding. The drainage route was cleaner than would be possible via a vaginal approach, possibly preventing postoperative retrograde re-infection.

**Conclusion:**

Laparoscopic surgery for postpartum retroperitoneal hematoma with infection was useful for both drainage and hemostasis.

## Highlights

• Laparoscopic treatment of a retroperitoneal postpartum hematoma with infection.
• Laparoscopy enabled sufficient drainage and surgical hemostasis of this hematoma.
• Laparoscopic drainage prevented retrograde re-infection via vaginal drainage.

## 1 Introduction

Delivery-related injuries to the blood vessels surrounding the birth canal can result in the formation of retroperitoneal hematomas. Such hematomas can spread throughout the retroperitoneum, resulting in hypovolemic shock, and hemostatic therapy via interventional radiology (IVR) and surgery has been reported [1]. Furthermore, such hematomas can lead to refractory infections, for which the optimal management strategies are unclear.

In a previous report of two cases of postpartum hematoma with infection, drainage was performed using a laparoscopic approach [2]. That procedure was especially useful in two ways: 1) as a diagnostic maneuver, to confirm the presence and location of the infected hematoma; and 2) as a treatment option for hemostasis and drainage in patients in whom conservative treatment failed. Here we present a similar case in which successful hemostasis and drainage was achieved via a laparoscopic approach. We also provide a surgical video.

The procedure was performed by a senior gynecologist after obtaining informed consent from the patient. This case report has been reported in line with the SCARE criteria [3]. The patient was managed in accordance with the principles laid down in the Declaration of Helsinki. Institutional review board approval was not required for this case report.

## 2 Case presentation

A primiparous in her 20s gave birth to a live female neonate (weighing 2,022 g) at 40 weeks’ gestation at an obstetrics clinic by vacuum-assisted delivery due to non-reassuring fetal status. She complained of abdominal pain and distension a few hours after delivery. Pelvic examination revealed no abnormalities and blood tests revealed only moderate anemia (hemoglobin concentration of 9.9 g/dL); as a precaution, the patient was placed under observation.

On postpartum day 2, her symptoms persisted, anemia worsened (hemoglobin concentration of 7.5 g/dL), and pelvic examination revealed a mass in the vaginal wall. The patient was transported to our hospital with a diagnosis of postpartum retroperitoneal hematoma. She had a history of asthma and allergies to pollen and house dust, and she was not taking medication at that point. She had no remarkable family history.

**Fig. 1:**
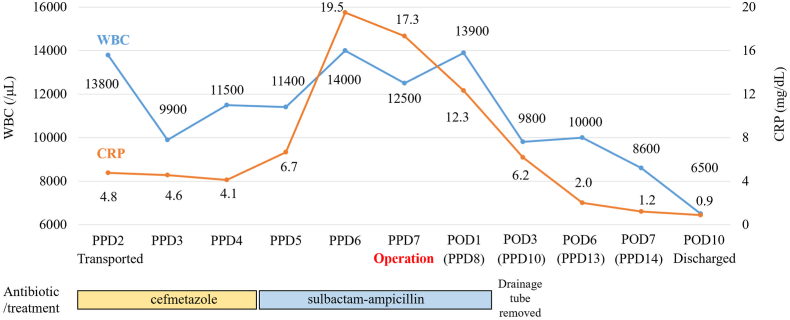
Case progression. The WBC counts, CRP level, and intervention (antibiotics and surgery) are provided. CRP: C-reactive protein, POD: postoperative day, PPD: postpartum day, WBC: white blood cell.

**Fig. 2:**
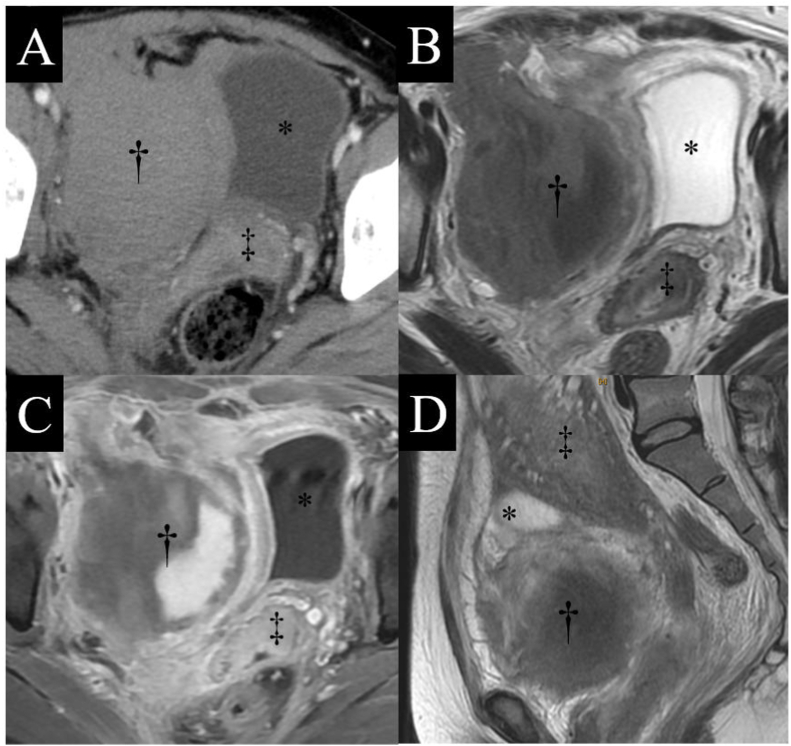
Preoperative images. Panels A–C are axial images of the positioning of the retroperitoneal hematoma and pelvic organs, and D is a sagittal image of the same. A) Contrast-enhanced computed tomography revealed no extravasation into the hematoma. B) and D) T2-weighted MRI revealed a hematoma on the right side of the pelvis, near the upper vaginal wall. C) Contrast-enhanced, T1-weighted MRI revealed a cystic hematoma with a contrast effect. The hematoma, uterus, and bladder are indicated as †, ‡, and *, respectively. MRI: magnetic resonance imaging.

Upon admission, her blood pressure, heart rate, body temperature, hemoglobin concentration, C-reactive protein (CRP) concentration, and white blood cell (WBC) count were 157/89 mmHg, 103 beats/min, 36.6 °C, 7.6 g/dL, 4.8 mg/dL, and 13,800/μL, respectively (Fig. 1). Upon pelvic examination, the sutured perineal incision was observed at 7 o'clock of the lower vagina with no hematoma at that site. Conversely, a tender hump was observed at 10 o'clock on the vaginal wall, on the right side of the uterine cervix. Ultrasonography revealed cystic lesions in the right side of the pelvis. Contrast-enhanced computed tomography (CT) revealed no active extravasation into the hematoma (Fig. 2A). Conservative antibiotics (cefmetazole) were initiated because hemostasis had already been achieved, there was no obvious infection, and drainage was considered anatomically difficult; however, the inflammatory response did not improve.

**Fig. 3:**
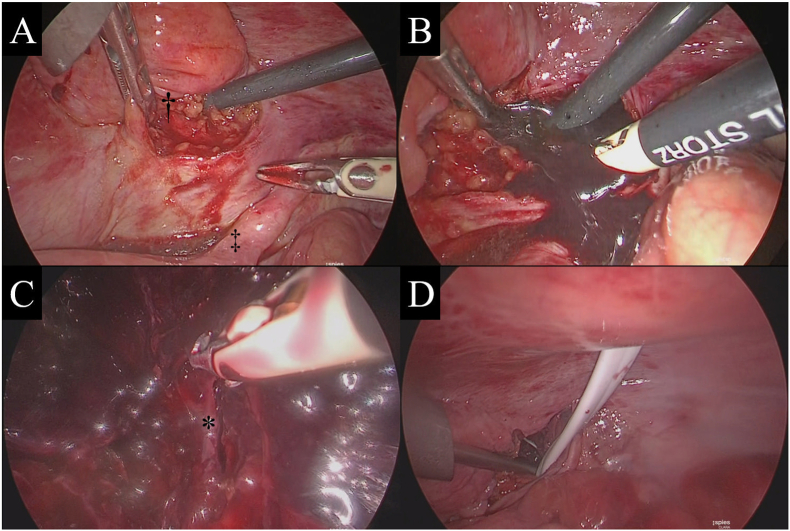
Operative findings. A-B) The peritoneum was incised to reach the lumen of the hematoma. The incision site on the surface of the hematoma and right round ligament are indicated as † and ‡, respectively. C) The pooled blood was removed, and the source of bleeding was identified. The bleeding vessel, identified as the vaginal vein, is indicated as *. D) A drainage tube was placed in the lumen of the hematoma.

On postpartum day 5, the patient developed a fever >38 °C, and the antibiotic was changed to sulbactam-ampicillin. On postpartum day 6, the fever still persisted, and the patient's CRP concentration and WBC count were 19.5 mg/dL and 19,489/μL, respectively. Magnetic resonance imaging (MRI) revealed a hematoma (80 × 70 × 63 mm) in the retroperitoneal space, expanding from the right paravaginal space to the right paravesical and parametrial spaces (Fig. 2B–D). As antibiotic therapy seemed ineffective, laparoscopy was performed on postpartum day 7. The operation was performed with the patient in the supine position under general anesthesia. Parallel ports were inserted on the right side to avoid the enlarged postpartum uterus. The right paravesical retroperitoneum was incised with monopolar electrosurgery. The connective tissue was dissected up to the hematoma, rupturing the cyst and releasing a fluid with dark red and muddy contents and clots, all of which was drained (Fig. 3A and B). After aspiration and washing of the fluid contents, direct laparoscopic observation of the inside of the hematoma revealed its expansion from the right paravesical space to the right paravaginal space. Moreover, small sites of bleeding from the right vaginal vein were observed. This bleeding was stopped with bipolar electric coagulation forceps (Fig. 3C). A drainage tube was placed in the dead space of the hematoma (Fig. 3D). The operation time was 90 min, and the volume of the aspirated hematoma was approximately 300 mL. There were no surgery-related complications.

After the operation, the inflammatory response decreased steadily, the negative pressure drainage tube was removed after 7 days, and the patient was discharged after 10 days.

At the 1-month postoperative visit, there were no abnormal blood tests and no pelvic mass on ultrasonography.

The surgical film of the operation is provided in Supplementary Video S1. The treatment course of the patient and antibiotics used are shown in Fig. 1.

Supplementary video related to this article can be found at https://doi.org/10.1016/j.amsu.2022.103686

The following is the supplementary data related to this article:

## 3 Discussion

We described a typical case of retroperitoneal postpartum hematoma with infection that was successfully drained and in which hemostasis was achieved via a laparoscopic approach. We also provided the surgical video of the procedure.

Postpartum hematomas most commonly develop in the birth canal, although paravaginal and vulvar hematomas can be large enough to reach the retroperitoneal space [4]. In asymptomatic cases, conservative treatment is preferred. Certain retroperitoneal hematomas require aggressive treatment for symptomatic relief, hemostasis, and infection control. For instance, when they are accompanied by antibiotic-resistant infection [5], as in our case, the clinician may decide on surgical management.

There has been a report of two cases of experimental laparoscopic surgery and drainage for postpartum hematoma with antibiotic-resistant infection [2]. The focal points of surgical management for retroperitoneal postpartum hematomas for which conservative treatment failed should be precise diagnosis, adequate hemostasis, and effective drainage without introducing retrograde infection. In this case, the diagnosis of retroperitoneal hematoma was simple because the size, location, and tenderness of the hematoma were suitable for pelvic examination, and we had access to MRI and contrast-enhanced CT. However, certain cases are more challenging to diagnose with conventional maneuvers [1]. In such cases, laparoscopy may be useful for diagnosis.

Retroperitoneal hematomas may be treated with different modalities. For patients with large hematomas, unstable vital signs, and active bleeding, IVR is recommended [1]. IVR has been recommended when conservative management is difficult, especially in cases of upper vaginal wall hematomas [1,6]. Drainage without complete hemostasis may result in loss of the tamponade effect and allow recurrence of the hematoma [1]. On the other hand, IVR is not available in all birth centers. Moreover, IVR for postpartum hematoma exposes the patient to radiation and may cause a decrease in blood supply to the ovaries and uterus of women of reproductive age [7]. Lower vaginal wall hematomas are usually drained transvaginally [1,5], but such drainage is more challenging for upper vaginal wall hematomas, owing to their anatomical location [8]. Transvaginal drainage also carries a risk of retrograde vaginal bacterial infection [8].

Considering the above, we chose the laparoscopic approach. The anatomical location of the hematoma in this case was a narrow space between the vaginal wall and pelvis, spreading into the paravesical space; hence laparoscopy was considered the safest approach. In a similar surgical case, Villette et al. [9] concluded that ovarian abscesses should be drained if there are no severe pelvic adhesions. In our case, there was no severe pelvic adhesion, and the patient had a stable general condition; hence, we deemed her suitable for laparoscopic surgery.

Adequate hemostasis was also achieved in our case with the laparoscopic approach. Laparoscopic surgery enables removal of blood and clots from the hematoma, as well as insertion of a scope and forceps into the hematoma. Pneumoperitonization enlarges the hematoma, providing a clear laparoscopic view for direct observation of its walls. The positive gas pressure also temporarily decreases active bleeding, simplifying identification of the source of bleeding. Subsequently, bipolar electrocoagulation forceps can be used to stop bleeding without damaging the surrounding organs. Finally, in this case, drainage was achieved with a tube guided percutaneously via the abdominal cavity from inside the hematoma. Compared to the transvaginal approach, a cleaner drainage route was provided, which might have prevented postoperative retrograde infection.

This intervention was beneficial in terms of infection and bleeding control.

However, patients with adhesions due to previous surgery or severe endometriosis may have difficulty reaching the hematoma and may require some case selection to perform this surgery. To the best of our knowledge, this report and that by Yokoe et al. [2] are the only case reports on the laparoscopic management of postpartum retroperitoneal hematomas complicated by infection; therefore, the data are too limited to determine whether laparoscopic surgery should be recommended for such cases. We eagerly await more detailed indications for surgical management as more cases are accumulated.

## 4 Conclusion

We demonstrated that laparoscopic surgery is a viable treatment option for postpartum retroperitoneal hematomas with antibiotic-resistant infections. This surgical strategy has the advantage of providing secure hemostasis and a clean drainage route.

## Ethical approval

Institutional review board approval was not required for this case report.

## Sources of funding

None to declare.

## Author contributions

KS, AY, and MM performed follow-up observation with antibiotics.

MK and KS performed the laparoscopic surgery.

TY conducted a literature search, prepared a surgical video and wrote the manuscript.

MK and HO contributed to the conception of the study.

All authors approved the final manuscript.

## Sources of funding

No funding was involved regarding this case report.

## Registration of Research Studies

Case reports are NOT first-in-man studies, do not need to be registered in Research Studies.

https://www.researchregistry.com/help-and-support/faqs.

## Guarantor

Takuya Yokoe, MD.

Department of Obstetrics and Gynecology, Kansai Medical University, 2-5-1 Sin-machi, Hirakata, Osaka 573-1191, Japan.

Tel.: +81-72-804-0101.

Fax: +81-72-804-2547.

E-mail: yokoetk@takii.kmu.ac.jp.

## Consent

Written informed consent was obtained from the patient for publication of this case report and accompanying images. A copy of the written consent is available for review by the Editor-in-Chief of this journal on request.

Patient perspective: “The content of informed consent was well understood. I do not mind my condition and treatment being addressed in the case study.”

## Provenance and peer review

Not commissioned, externally peer-reviewed.

## Declaration of competing interest

None to declare.
